# A 30-year-old male with innumerable millimetric pulmonary nodules: tunnelling thoughts

**DOI:** 10.1016/j.rmcr.2026.102461

**Published:** 2026-07-11

**Authors:** Delian E. Hofman, Aldo Tanushi, Frits M.E. Franssen, Lisa M. Hillen, Marijke Rutten

**Affiliations:** a Department of Respiratory Medicine, Maastricht University Medical Center, Maastricht, the Netherlands; b Department of Pathology, GROW-School for Oncology and Reproduction, Maastricht University Medical Center, MUMC, Maastricht, the Netherlands

**Keywords:** Clinical respiratory medicine, Environmental & occupational health and epidemiology, Pathology, Radiology and other imaging, Tuberculosis

## Abstract

We report the case of a young Syrian male who presented to the emergency ward with mild exertional dyspnea and centrilobular millimetric pulmonary nodules. Given the broad differential and inconclusive medical history, a comprehensive diagnostic work-up was undertaken. Bronchoscopy with lymph node biopsy revealed a non-specific granuloma, while concurrent infectious screening identified tuberculosis infection (TBI). Further assessment of his occupational history revealed tunnelling work and raised a clinical suspicion for silicosis, an interstitial lung disease caused by the inhalation of crystalline silica dust, which was confirmed by pulmonary biopsy.

## Highlights

• Early-stage silicosis can present with innumerable millimetric pulmonary nodules and may be underrecognized in routine clinical practice.
• A detailed anamnesis on occupational, recreational, and environmental exposure is crucial to recognize silicosis early.
• Physicians should screen for TB disease and TBI in patients with silicosis to prevent further disease deterioration due to pulmonary TB disease.

## 1 Introduction

The differential diagnosis of diffuse pulmonary nodules in patients with respiratory complaints is extensive. It encompasses infectious diseases, malignancies, and interstitial lung diseases (ILDs). Diagnosing can be challenging but is essential, as treatment and prognosis vary widely. A thorough clinical history, including occupational and environmental exposures, plays a crucial role in guiding the diagnostic process and avoiding misdiagnosis. This case report describes the case of a young Syrian male who presented to the emergency ward with mild exertional dyspnea and diffuse millimetric pulmonary nodules.

## 2 Case report

A 30-year-old Syrian male was admitted from the emergency department of Maastricht University Medical Center in the Netherlands with a one-day history of mild exertional dyspnoea and epigastric and dorsobasal thoracic pain which worsened with respiration. The patient moved from Syria to Lebanon four years ago and migrated to the Netherlands two months before presentation with a transit through Turkey. The subsequent route to the Netherlands remained unclear due to human trafficking. His medical history included a hospital admission in Syria six years ago for a respiratory infection of uncertain cause. He was a current smoker with twelve pack years. The patient had no history of tuberculosis (TB). He had no symptoms of haemoptysis, fever, weight loss, joint pain, or skin rash. His family history was unremarkable.

At presentation, the patient was eupnoeic with a peripheral oxygen saturation of 98% in ambient air, had an afebrile temperature, a regular heart rate of 91 beats/min, and blood pressure of 113/63 mmHg. Chest auscultation revealed no abnormal findings. Percussion over the left basal chest, both dorsal and anterior, was painful. Physical examination of the extremities did not show any signs of deep venous thrombosis. The patient's extremities did not show clubbing and no skin lesions were observed.

Laboratory findings showed no abnormalities in liver- and renal function and complete blood count. D-dimers were 3400 ng/ml (normal level: <500 ng/ml) and C-reactive protein level was 48 mg/L (normal level: <10 mg/L). Calcium level was 2.38 mmol/L corrected (normal level: 2.2-2.7 mmol/L). Electrocardiogram and chest radiography (Fig. 1A+B) showed no abnormalities. A chest CT scan with pulmonary embolism protocol ruled out pulmonary embolism but showed innumerable bilateral millimetric pulmonary nodules with centrilobular distribution and bilateral hilar lymphadenopathy (Fig. 2), findings that were also present after one week of admission on a high-resolution chest CT (HRCT) (Fig. 3A+B). A comprehensive occupational and environmental history was obtained at admission, which revealed previous plumbing work but no apparent relevant exposures. The history was taken with the assistance of an independent medical interpreter, however, communication remained challenging, partly due to language barriers.

**Fig. 1:**
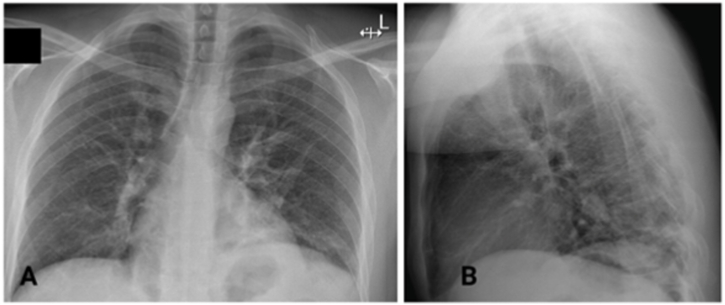
A + B. Anterior-posterior (A) and lateral (B) radiograph of the thorax showing no abnormalities.

**Fig. 2:**
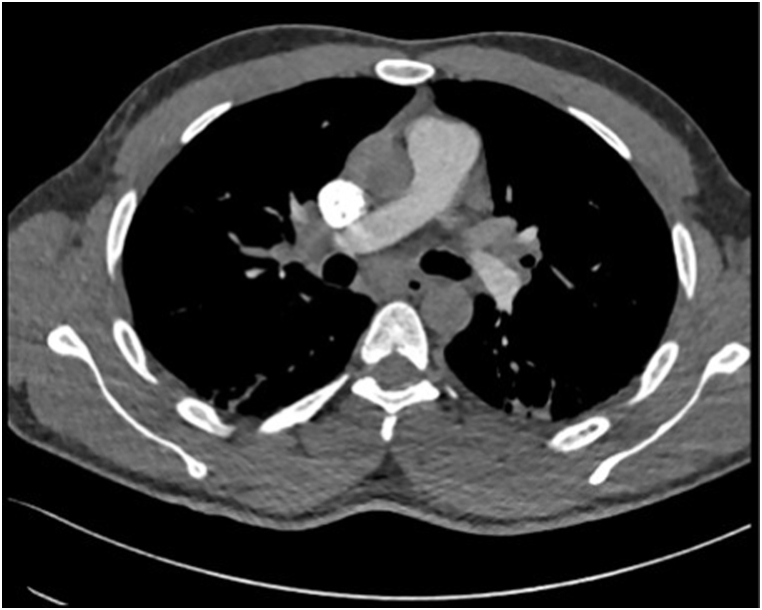
Transversal chest CT scan with pulmonary embolism protocol showing bilateral hilar lymphadenopathy.

**Fig. 3:**
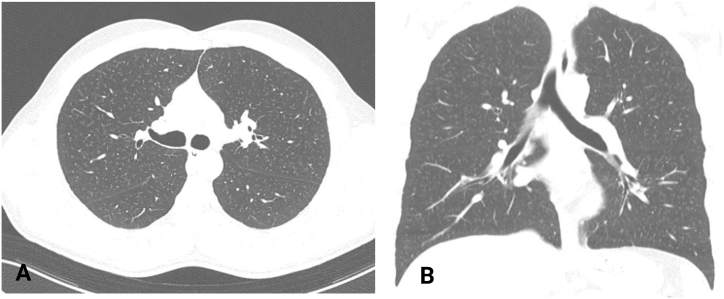
A + B. Transversal (A) and frontal (B) high-resolution computed tomography (HRCT) showing innumerable pulmonary nodules distributed in a centrilobular pattern.

Subsequently, bronchoscopy with bronchoalveolar lavage (BAL) was performed and cytology contained macrophage levels of 95.8%. Iron stain and acid-fast stain of BAL fluid was negative. Cytology of endobronchial ultrasound (EBUS) with thoracic lymph node biopsy of N7 and N11L revealed a dubious granuloma. Infectious workup was performed, including Gram- and auramine staining, (mycobacterial) cultures, and TB polymerase chain reaction (PCR) of lymph node biopsy, BAL fluid, and urine. However, no micro-organisms were identified and PCR TB on lymph node biopsy and BAL fluid was negative. Additional HIV and hepatitis A, B, and C blood testing was negative. Interferon-Gamma Release Assay (IGRA) testing was positive with TB1 and TB2 levels of 0.37 and 0.45 IU/mL, respectively. Test results ≥0.35 IU/mL were considered positive. Ultimately, video-assisted thoracoscopy (VATS) with lung biopsy of the right upper, mid, and lower lung was performed, as other diagnoses, including sarcoidosis or malignancy, could not be conclusively ruled out. The histopathology showed a mixed-dust pathology with silicosis (Fig. 4 A to F and Fig. 6). Additional staining, including Gram-, Grocott-, Periodic Acid-Schiff-, and Ziehl-Neelsen, yielded negative results. TB PCR and mycobacterial cultures of the VATS biopsies were negative. Pulmonary function testing showed no signs of obstructed or restricted pulmonary function and diffusing capacity of the lungs for carbon monoxide (DLco) was normal (95% of predicted). During the diagnostic work-up, the patient presented worksite photographs (Fig. 5) that clarified his occupational history, confirming approximately four years of employment in tunnelling, as well as various construction roles with dust exposure from sawing and sanding marble and other natural stone. In all occupancies, no respiratory protection was provided. After discharge, the patient was treated for tuberculosis infection (TBI), previously known as “latent TB infection”, with a three-month course of isoniazid and rifampicin [1]. Furthermore, the patient was given the advice to quit smoking and to avoid additional exposure to silica or to use respiratory protective measures, which he was adherent to. Chest radiography and lung function remained stable remained stable at 3-, 6-, and 12-month follow-up at the pulmonary outpatient clinic.

**Fig. 4:**
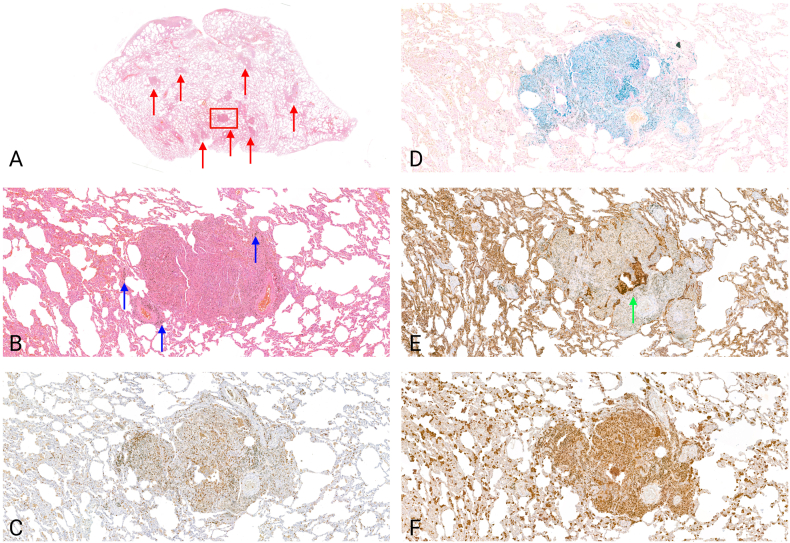
A-F. Histopathology of lung biopsy showing associated findings. Legend: Haematoxylin and eosin (H&E) staining reveals centrilobular nodules (A, magnification 0,8x, red arrows) with anthracotic pigmentation when zooming in to a 1.55 mm nodule (B, magnification 10x, blue arrows). CD3^+^ T-cells and pigmented macrophages are visible in CD3 staining (C, magnification 10x). Iron (FE) staining reveals hemosiderine deposition (D, magnification 10x). CK7 staining reveals a bronchiolocentric distribution of the noduli, indicative of pneumoconiosis (E, magnification 10x). Numerous macrophages are shown by CD68 staining (F, magnification 10x).

**Fig. 5:**
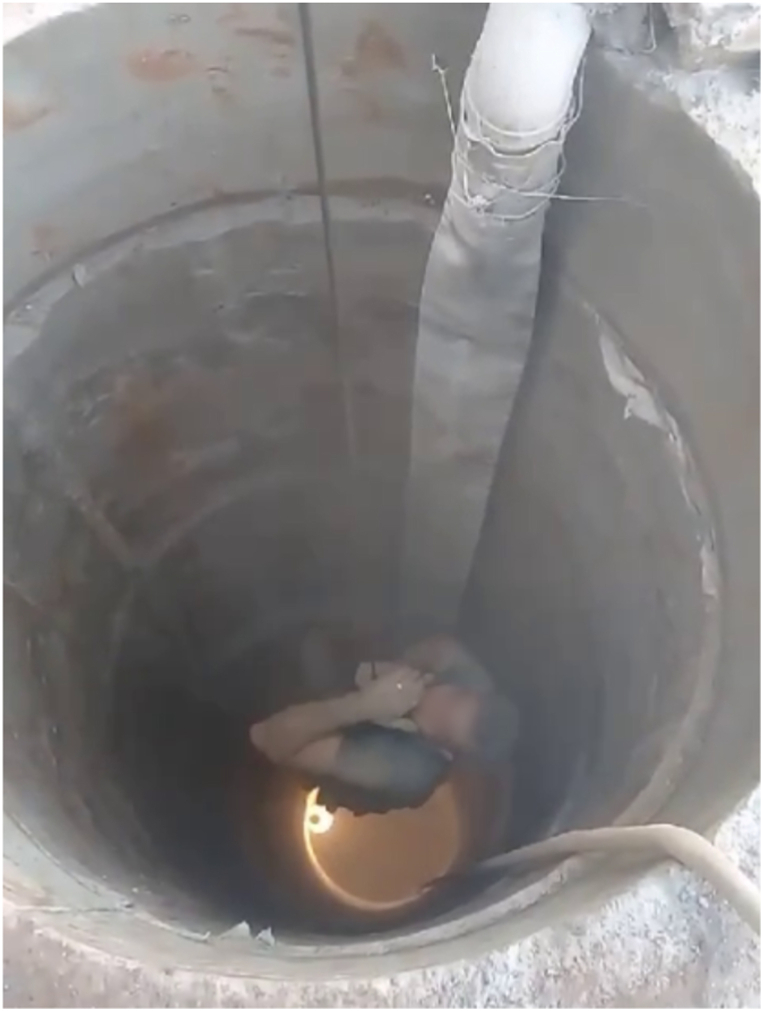
A 30-year-old Syrian male working in the tunneling industry in Lebanon.

**Fig. 6:**
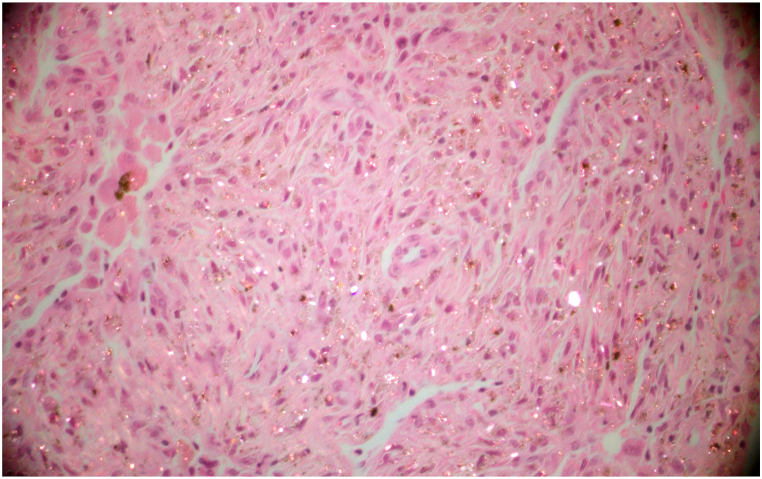
Polarized light microscopy of the lung biopsy. Legend: Polarized light microscopy showing strong birefringent anorganic particles appearing as bright, irregularly shaped or needle-like structures within the macrophages and associated with collagen bundles in the nodular fibrotic lesions (magnification 40x).

## 3 Discussion

Silicosis is an irreversible form of ILD, caused by inhalation of respirable crystalline silica (RCS) dust. RCS exposure often occurs in labour across various industries, including construction work, stonemasonry, and mining [2,3]. Silicosis can mimic many other pulmonary diseases, presenting with millimetric pulmonary nodules on imaging in early stages. Therefore, early-stage silicosis can be difficult to diagnose and is sensitive to diagnostic delays or even misdiagnosis.

A detailed anamnesis on medical history related to the duration and intensity of occupational, environmental, or other exposures, is crucial in patients experiencing (unexplained) respiratory complaints. The primary method of diagnosis and classification of silicosis consists of chest radiography, in combination with patient history, as defined by the International Labour Organization (ILO) [4]. However, no abnormalities were seen on radiography in our patient and previous studies have shown that HRCT might be more sensitive than is conventional radiography in the detection of silicosis, particularly in detecting early lung lesions [5,6]. The extensive diagnostic work-up of this case was mandated by the patient's relatively young age, initial non-revealing employment history, and vague general symptoms at presentation. These factors led to a wide differential, including sarcoidosis, other ILDs, such as respiratory bronchiolitis-ILD (RB-ILD) given the patient's smoking history and imaging findings, and malignancy. While often not performed, polarized light microscopy was used here to detect crystalline silica (Fig. 6).

In this case, the patient was found to have TBI without active disease. Although IGRA testing was marginally positive, the patient was preventatively treated for TBI. This decision was made based on WHO guidelines on preventive treatment of tuberculosis [7], as the combination of silicosis and TBI increases the likelihood of developing active TB, possibly due to impaired activity of alveolar macrophages [8]. Systematic screening of TB is recommended by the WHO for all patients with silicosis as they are at increased risk of having mycobacterial diseases [[8], [9], [10]]. Notably, active smoking increases the risk of TBI and development of active TB disease in individuals with silicosis. As both smoking and TB are independently associated with lung function impairment [11], cessation of smoking should be advised in any case.

Although prevention efforts have been made by the ILO/WHO Global Programme for Elimination of Silicosis (GPES), silicosis remains prevalent with 23,695 incident cases described globally in 2017 [12]. Additionally, recent case series have reported outbreaks of acute and accelerated silicosis in modern occupations [[13], [14], [15]]. This is concerning as these patients present with shorter disease latencies and have poorer prognoses. Further efforts in raising global awareness for the occupational health hazards of silica through ongoing and updated prevention- and dust monitoring programs would enable us to gain recognition and control of this primarily preventable, often progressive, and life-threatening disease [16].

In conclusion, this case underscores the critical role of thorough occupational history-taking in the diagnostic work-up of patients with diffuse pulmonary nodules, particularly in displaced individuals with incomplete histories.

## Ethics and consent statement

The authors declare that written informed consent was obtained for the publication of this manuscript and accompanying images and attest that the form used to obtain consent from the patient(s) complies with the Journal requirements as outlined in the author guidelines.

## Funding statement

Not applicable.

## CRediT authorship contribution statement

**Delian E. Hofman:** Project administration, Visualization, Writing – original draft, Writing – review & editing. **Aldo Tanushi:** Investigation, Writing – review & editing. **Frits M.E. Franssen:** Supervision, Writing – review & editing. **Lisa M. Hillen:** Investigation, Supervision, Writing – review & editing. **Marijke Rutten:** Conceptualization, Supervision, Validation, Visualization, Writing – review & editing.

## Declaration of competing interest

The authors declare the following financial interests/personal relationships which may be considered as potential competing interests: D.E.H., A.T., and M.R. have no conflicts of interest to declare. L.M.H. reports holding licenses with NVVP, IAP, and Deutsche Ärztekammer. F.M.E.F reports consulting fees from Sanofi and MSD, payment or honoraria for lectures, presentations, speakers bureaus, manuscript writing or educational events from AstraZeneca, Chiesi, GSK, Sanofi, and Pfizer and support for attending meetings and/or travel from AstraZeneca.
